# A Surgical Cryoprobe for Targeted Transcorneal Freezing and Endothelial Cell Removal

**DOI:** 10.1155/2017/5614089

**Published:** 2017-05-16

**Authors:** Alina Akhbanbetova, Shinichiro Nakano, Stacy L. Littlechild, Robert D. Young, Madara Zvirgzdina, Nigel J. Fullwood, Ian Weston, Philip Weston, Shigeru Kinoshita, Naoki Okumura, Noriko Koizumi, Andrew J. Quantock

**Affiliations:** ^1^Structural Biophysics Research Group, School of Optometry and Vision Sciences, Cardiff University, Maindy Road, Cardiff CF24 4HQ, UK; ^2^Department of Biomedical Engineering, Faculty of Life and Medical Sciences, Doshisha University, 1-3 Miyakodami-Tatara, Kyoto 610-0321, Japan; ^3^Division of Biomedical and Life Sciences, Faculty of Health and Medicine, Lancaster University, Lancaster LA1 4YQ, UK; ^4^Network Medical Products Ltd. Coronet House, Kearsley Road, Ripon, North Yorkshire HG4 2SG, UK; ^5^Department of Frontier Medical Science and Technology for Ophthalmology, Kyoto Prefectural University of Medicine, Hirokoji-Kawaramachi, Kyoto 602-0841, Japan

## Abstract

**Purpose:**

To examine the effects of transcorneal freezing using a new cryoprobe designed for corneal endothelial surgery.

**Methods:**

A freezing console employing nitrous oxide as a cryogen was used to cool a series of different cryoprobe tip designs made of silver for high thermal conductivity. In vitro studies were conducted on 426 porcine corneas, followed by preliminary in vivo investigations on three rabbit corneas.

**Results:**

The corneal epithelium was destroyed by transcorneal freezing, as expected; however, the epithelial basement membrane remained intact. Reproducible endothelial damage was optimally achieved using a 3.4 mm diameter cryoprobe with a concave tip profile. Stromal edema was seen in the pre-Descemet's area 24 hrs postfreeze injury, but this had been resolved by 10 days postfreeze. A normal collagen fibril structure was seen 1 month postfreeze, concurrent with endothelial cell repopulation.

**Conclusions:**

Transcorneal freezing induces transient posterior stromal edema and some residual deep stromal haze but leaves the epithelial basement membrane intact, which is likely to be important for corneal re-epithelialization. Localized destruction of the endothelial monolayer was achieved in a consistent manner with a 3.4 mm diameter/concave profile cryoprobe and represents a potentially useful approach to remove dysfunctional corneal endothelial cells from corneas with endothelial dysfunction.

## 1. Introduction

Corneal transparency is maintained in the healthy eye by a monolayer of endothelial cells on the inner surface of the cornea. Even though human corneal endothelial cells do not possess the capacity for proliferation in vivo, the endothelium as a whole has a functional reserve to cope with cell loss via the spreading and enlargement of cells adjacent to those lost [1, 2]. Excessive endothelial loss and deterioration caused by eye pathologies such as Fuchs' endothelial corneal dystrophy (FECD), however, lead to corneal edema, clouding, and eventually loss of vision. FECD is a progressive degenerative disorder that is a major indication for corneal transplant surgery. Surgical intervention in the form of a full-thickness-penetrating keratoplasty—or more commonly nowadays a posterior lamellar graft—is the main treatment option. But, despite the success of corneal graft surgery, some questions about the long-term survival of the donor tissue [3] and the recurring problem of sufficient tissue availability remains. These limitations have led researchers to seek potential alternatives to corneal transplantation to treat corneal endothelial dysfunction.

One promising route involves the use of selective inhibitors of the Rho kinase pathway. The so-called ROCK inhibitors regulate the actin cytoskeleton and influence vital cell activities such as motility, proliferation, and apoptosis [4]. Owing to their demonstrable value, numerous studies have been conducted in recent years which focus on the effect of ROCK inhibitors on corneal endothelial cells both in vivo and ex vivo [5–9]. One approach involves transcorneal freezing to damage corneal endothelial cells in the central portion of the cornea in patients with FECD followed by the topical delivery of a ROCK inhibitor, Y27632, in the form of eye drops to encourage peripheral endothelial cells that had been unaffected by the freeze injury to repopulate the central zone of the corneal endothelium [10–12]. Freeze damage is achieved by application of a cold probe to the corneal surface. Another approach involves cell injection therapy whereby cultivated human corneal endothelial cells are injected into the anterior chamber of the eyes with FECD in a suspension that includes Y27632 ROCK inhibitor [13]. This agent has also been tested in the form of eye drops as a long-term pharmacological treatment for bullous keratopathy [14].

A small series of first-in-man surgeries to test the concept of transcorneal freezing followed by short-term ROCK inhibitor eye drop application for the treatment of FECD was conducted a few years ago and showed promise [10–12]. In this approach, the tip of a stainless steel rod, 2 mm in diameter, was immersed in liquid nitrogen at −196°C before being applied to the surface of the central cornea for an arbitrarily chosen time of 15 sec. The assumption was that central corneal endothelial cells located underneath the cold-rod applicator would be destroyed by freeze injury, although this could not be directly confirmed in the human subjects because the cloudy FECD corneas did not allow a view of the endothelium by specular microscopy. The freezing of corneal tissue has also been used as a modality to induce an injury to facilitate basic research into corneal wound healing [15–28]. If corneal freezing is to be used in a clinical setting, however, (either for the destruction of diseased cells in the central endothelium prior to ROCK inhibitor eye drop application for FECD as described above or to pretreat the cornea prior to targeted drug delivery to combat conditions such as fungal keratitis) we contend that it needs to be achieved in a more sophisticated, reliable, and reproducible manner than that achieved with an immersion-cooled steel rod. Here, we report the development and validation of a new cryoprobe based on the expansion of nitrous oxide as a cryogen and its effect, in vitro and in vivo, on the corneal epithelium, stroma, and endothelium.

## 2. Materials and Methods

### 2.1. Cryoprobe Development

**Figure 1:**
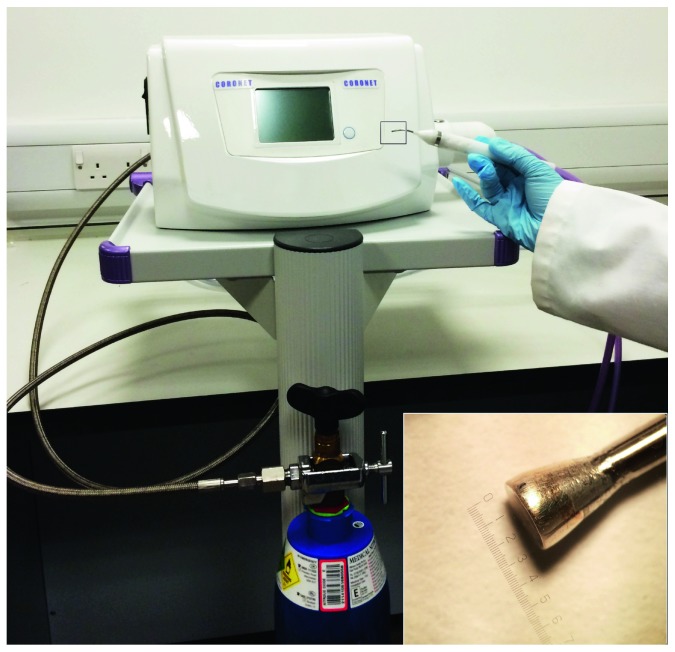
The transcorneal freezing machine attached to a cylinder of medical grade nitrous oxide (the cryogen), which comprises a main console with a monitor screen that illustrates freeze time, cryogen levels, and readiness for freeze plus interchangeable probe tips and a footswitch (not shown). Inset: the 3.4 mm diameter concave probe tip manufactured from silver for high thermal conductivity.

A console that uses nitrous oxide as a cryogen was manufactured in conjunction with a series of cryoprobes with newly designed tips, some of which matched the cornea's curvature (Figure 1). This prototype project was carried out by Coronet Medical Technologies Ltd., the ophthalmic arm of Network Medical Products Ltd. Enclosed gas expanded within the tip and was recycled therein, achieving a low temperature based on the Joule/Thomson effect. The tip of the cryoprobe, used to contact the corneal surface, was circular around the probe's main axis and a number of designs were tested. Probe tips were 1.8 mm, 2.4 mm, or 3.4 mm in diameter and were manufactured from silver for high thermal conductivity. Larger diameter probe tips were not considered because of the option of multiple surface freeze placements should a wider area of the cornea need to be treated. Probes had either a flat surface profile or a concave one with a radius of curvature of 8 mm. For ease of use, a foot switch was incorporated into the design, which initiates cooling at the cryoprobe tip and maintains the reduced temperature throughout the whole time it is depressed. The foot switch is linked to a timer on the main console that provides a visual output of freezing time plus an audible signal (with a mute option) at 1 sec intervals when the foot switch is depressed. Freezing temperature at the probe tip (−50°C) is reached within 2 sec of depressing of the foot switch; after release, ambient room temperature is achieved within seconds. The hand-held cryoprobe has an ergonomic-angled design to allow easy application to the corneal surface (Figure 1). Probes should be thoroughly cleaned, inspected, and autoclaved prior to use.

### 2.2. Transcorneal Freezing In Vitro

The porcine cornea is comparable to that of the human cornea in terms of its structure and its overall dimensions and the pig eye is thus often used for practice by trainee corneal surgeons. The central corneal thickness in adult pigs is usually around 660 *μ*m [29, 30], which approximates a representative measurement of edematous corneal thickness in individuals with FECD [31, 32]. The porcine eyes, therefore, were well suited for our investigations, and the intact eyeballs, including extraocular muscles, were obtained soon after slaughter at a local abattoir (W. T. Maddock, Kembery Meats, Maesteg, Wales, UK). These were brought to the laboratory on ice and experiments were begun within 2-3 h of death. When the eyes arrived at the home laboratory, ultrasound measurements of central corneal thickness were made with a Tomey SP-100 pachymeter (Erlangen, Germany), which revealed that the corneas had thickened (~1000 *μ*m) postmortem compared to those of published values [31, 32]. Consequently, the eyes were placed in a humidified incubator (Brinsea Octagon 100, Egg Incubator, Sandford, UK) at 45°C for 30–45 min to reverse the postmortem swelling and attain a thickness similar to what might be expected in humans with FECD. In total, 426 porcine eyes were used for the in vitro experiments in which the corneal thickness ranged from 483 *μ*m to 831 *μ*m owing to differences in eye size and likely differential postmortem swelling and deswelling.

Immediately after an eye was removed from the humidified incubator, its central corneal thickness was recorded as an average of eight measurements. The cryoprobe tip was then applied to the corneal surface and cooling was activated by pressing the foot switch to control the flow of nitrous oxide gas. Freezing times of 3, 5, 9, and 13 sec were tested, allowing an additional 2 sec for the freezing temperature to be reached in all cases (thus, a 3 sec freeze required for a 5 sec application and foot-switch depression). The cornea was then carefully excised at the limbus after which staining solutions of 0.2% alizarin red (~1 ml) and 0.25% trypan blue were applied sequentially to the endothelial surface for 60–90 sec each to identify dead cells. The stains were then gently washed off using approximately 5 ml of 0.9% sodium chloride buffer solution, after which digital images of the corneal endothelial surface were captured on a Zeiss Stemi 1000 light microscope (Carl Zeiss, Jena, Germany). A successful freeze injury was deemed to have occurred when a clear circular wound area was seen. The endothelial wound area in each corneal image was manually traced and calculated using Image J software (http://imagej.nih.gov/ij/). Magnification was calibrated for each set of experiments using the image of an eyepiece graticule. Averages of three measurements were calculated and data were further collated using Microsoft Excel software (Microsoft Corp., Redmond, WA, USA).

### 2.3. Statistical Analysis

Statistical analyses were carried out operating IBM SPSS Statistics software (Version 23.0, IBM Corporation, New York, USA). Spearman's rank order correlation test was run to determine the relationship between central corneal thickness and endothelial wound areas induced by the freezing.

### 2.4. Scanning Electron Microscopy

To investigate the endothelial damage morphologically, four treated corneas were examined by scanning electron microscopy (SEM). Immediately after excision, the corneas were fixed in 2.5% glutaraldehyde and 2% paraformaldehyde in 0.1 M sodium cacodylate buffer, pH 7.3, for at least 2 h. Samples were then washed in PBS followed by the gradual dehydration through a graded ethanol series (from 50% to 100% in 30 min steps), after which the ethanol was replaced by hexamethyldisilazane for 20 min to minimize the shrinkage of the specimens. After drying, the corneas were placed on stubs (Agar Scientific, Stansted, UK) and sputter coated with gold (Edwards S150 sputter coater, Edwards High Vacuum Co. International, Wilmington, USA) to allow imaging in a JEOL JSM 5600 scanning electron microscope (JEOL Company, Tokyo, Japan) operating with a beam acceleration voltage of 15.0 kV.

### 2.5. Transcorneal Freezing In Vivo

Three adult New Zealand White rabbits were used to investigate corneal recovery after transcorneal freezing. At all stages, animals were treated in accordance with the ARVO Statement for the Use of Animals in Ophthalmic and Vision Research, and the research was approved by the IRB of Doshisha University. As reported later, the 3.4 mm concave cryoprobe was found to be the most effective in inducing endothelial damage in the porcine eye in vitro, but owing to the thinner cornea of the rabbit, the 2.4 mm concave cryoprobe was chosen for the in vivo transcorneal freezing experiments.

Under general anesthesia, a 2.4 mm probe was applied to the corneal surface of the rabbit eyes for a total of 5 sec. The contralateral eye was used as a control. Transcorneal freezing did not induce any severe general adverse effects. After 24 h, 10 days, and 1 month of treatment, the anterior segment of each eye was assessed by the use of a slit-lamp microscope and the rabbits were then euthanized. Corneal thickness was determined by the use of an ultrasound pachymeter (SP-2000; Tomey, Nagoya, Japan), and the mean of eight measured values was calculated. Intraocular pressure was measured by the use of a Tonovet tonometer (292000; KRUUSE, Langeskov, Denmark). Transmission electron microscopy (TEM) was conducted, as described below, on the corneas of the rabbits that were euthanized at 24 h- and 1-month time points after transcorneal freezing.

### 2.6. Transmission Electron Microscopy

The rabbit corneas were examined by TEM 24 h and 1 month postfreeze was conducted in Doshisha University, Japan. Briefly, after animals were euthanized, corneas were excised at the limbus and fixed in 2.5% glutaraldehyde and 2% paraformaldehyde in 0.1 M Sörensen buffer (pH 7.2–7.4) overnight at 4°C. Samples in fresh fixative were then express shipped to the UK. Full-thickness-dissected portions of the corneas were then subjected to alcohol dehydration and resin infiltration, after which they were embedded in epoxy resin (Araldyte CY212 resin, TAAB Laboratories, England, UK). Ultrathin sections were stained with uranyl acetate and lead citrate and examined on a JEOL 1010 microscope operating at 80 kV (JEOL Company, Tokyo, Japan).

## 3. Results

### 3.1. In Vitro Transcorneal Freezing

Initial experiments into the degree of endothelial damage caused by transcorneal freezing induced by four different types of cryoprobe tip—that is, 1.8 mm diameter/flat profile, 2.4 mm diameter/flat profile, 2.4 mm diameter/concave profile, and 3.4 mm diameter/concave profile—revealed that the time of contact with the corneal surface did not affect the area of endothelial damage, with freezing times of 3, 5, 9, and 13 sec tested (data not shown). Based on this outcome, a freezing time of 3 sec was chosen for all the in vitro experiments described herein. It also became apparent during the initial investigations that the probe tip had to be in contact with the corneal surface before the foot switch was depressed to initiate cooling. If the probe tip was cooled in air prior to being brought into contact with the cornea, no appreciable endothelial damage was seen; no matter how long, up to 15 sec, the probe remained in contact with the cornea.

**Figure 2:**
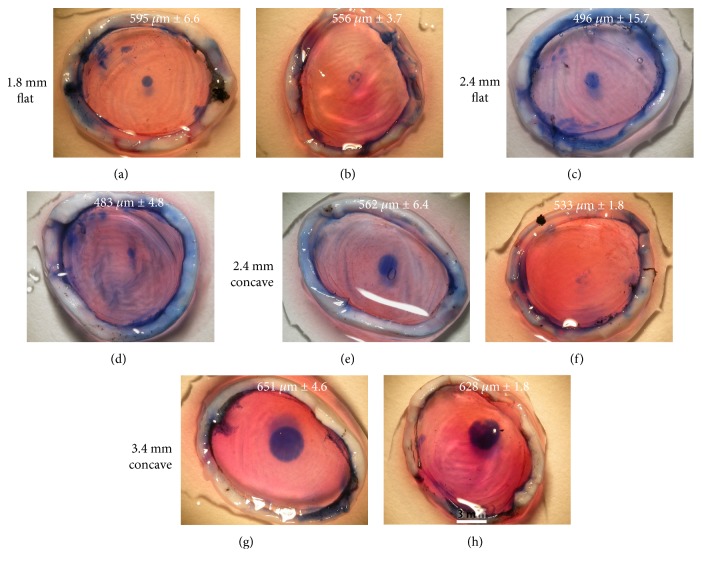
Representative images of corneal endothelial freeze injury on the pig eyes ex vivo induced by 3 sec freeze with four different cryoprobe tips and assessed by trypan blue staining. The thickness of each cornea is indicated on each panel (±SD) based on eight pachymetry readings. The area of cell damage is seen via the blue stain, and successful and non/less successful freeze injuries are shown in the left and right columns, respectively. Freezing with the 1.8 mm diameter/flat profile probe only rarely resulted in a reproducible wound (a and b). Endothelial freeze injury was more reliably achieved with 2.4 mm diameter probe tips with flat or concave profiles (c–f), but the optimal result and best consistency was achieved using the 3.4 mm diameter/concave profile cryoprobe (g and h). Scale bar, 3 mm. See Table 1 also.

Light microscopy of the endothelial surfaces of the postfreeze, trypan blue-stained corneas indicated the area of cell damage caused by the four different probes used in these experiments. Representative images of 426 technical replicates denoting the typical extent of endothelial damage are shown in Figure 2, with examples of what were considered to be successful or unsuccessful freeze injuries. A successful freeze injury was defined as one that resulted in a well-delineated circular area of cell damage. An unsuccessful freeze injury, on the other hand, was considered to have occurred either when there was no evidence of any cell damage or when the area of damage was irregular. Based on these criteria, only 14 of 102 eyes (14%) treated with the 1.8 mm diameter/flat profile probe were judged to have been successfully wounded, whereas 78 of 108 (72%) treated with the larger (i.e., 2.4 mm diameter) flat profile probe contained successful endothelial injuries. A similar number of the eyes exhibited corneal endothelial freeze damage—that is, 76 eyes of 108 (70%)—when the 2.4 mm diameter cryoprobe with a concave profile was used. Our data clearly indicated, however, that the 3.4 mm diameter/concave profile cryoprobe induced the most consistent endothelial damage with 90 eyes of 108 (83%) being successfully wounded in a reproducible manner (Table 1).

To quantify the extent of endothelial cell damage, we used Image J to manually trace around each wound deemed to have been successfully created (i.e., *n* = 258 of 426 technical replicates). The area of each wound was calculated, which, unsurprisingly, disclosed that larger probe tips led to more extensive endothelial damage (Table 1). All of the 426 corneas examined were subjected to multiple pachymetry measurements immediately prior to transcorneal freezing. This revealed that the average corneal thickness was 649 *μ*m (±61 *μ*m SD), which is a fair representative value for corneal edema in humans with endothelial dysfunction [31, 32]. Moreover, when the corneal thickness of the individual corneas was taken into account, a statistical Spearman's rank order correlation test identified that there was a weak relationship between central corneal thickness and endothelial damage when treated with the smallest and largest probes (1.8 mm diameter (*r* = –0.208, *n* = 102, *p* = 0.408) and 3.4 mm diameter (*r* = –0.258, *n* = 108, *p* = 0.007) but a stronger relationship using the 2.4 mm diameter probe tips with concave and flat profiles (*r* = –0.433, *n* = 108, *p* < 0.001; 2.4 mm (*r* = –0.466, *n* = 108, *p* < 0.001, respectively) (Table 1).

**Figure 3:**
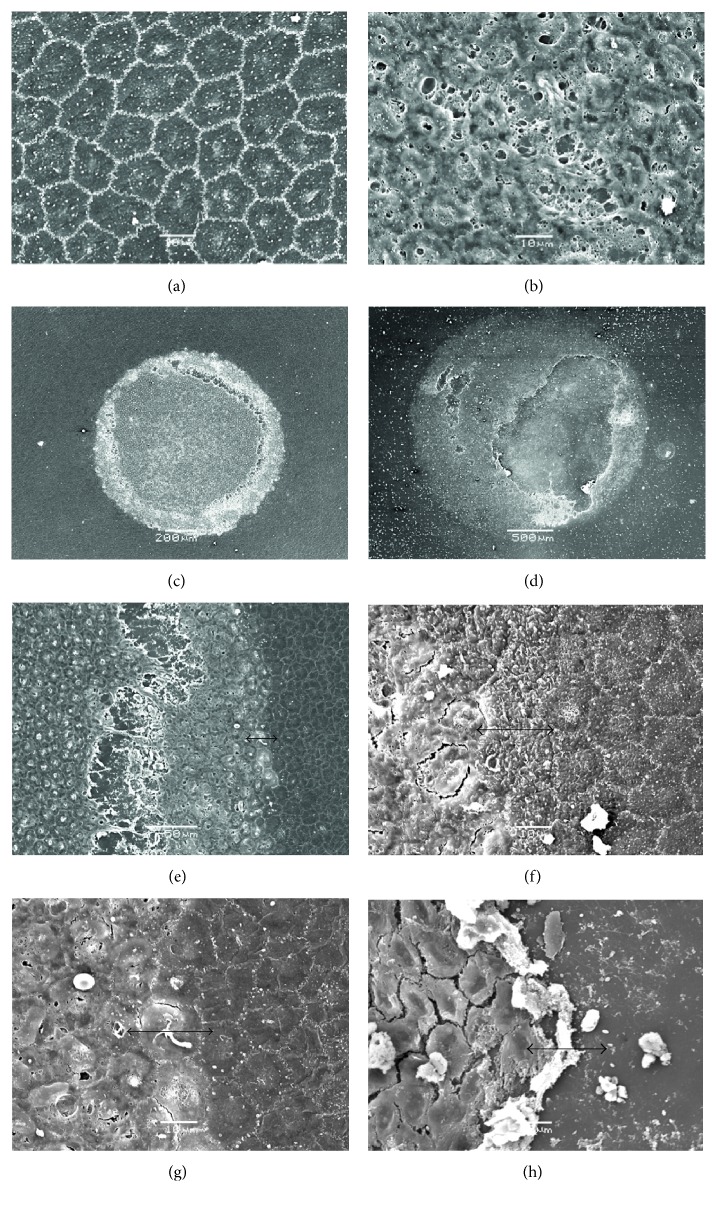
SEM of the endothelium of transcorneally frozen pig corneas. (a) An untreated pig cornea with cell boarders in white showing a characteristic hexagonal mosaic. The outline if the cell nucleus is evident as a slightly lighter area within each cell. Scale bar, 10 *μ*m. (b) A representative image taken at the same magnification of the freeze-damaged area after treatment with a 2.4 mm diameter/concave profile tip, illustrating severe damage to endothelial cells by freezing. Scale bar, 10 *μ*m. (c and d) Lower magnification images of endothelial freeze-injured wounds showing circular areas of endothelial cell damage including some endothelial debridement, exposing Descemet's membrane (c): 2.4 mm diameter/concave profile cryoprobe (scale bar, 200 *μ*m); (d): 3.4 mm diameter/concave profile cryoprobe (scale bar, 500 *μ*m). As expected, the larger probe induces more widespread damage (see Table 1 also). (e–h) Transition zones between unfrozen endothelial cells and those that were destroyed by freeze injury are often sharp (e) and (g); same area but different magnification (scale bars,10 *μ*m, apart from (g) which is 50 *μ*m).

To provide higher resolution information as to the status of the cells after freeze injury in the in vitro pig eye model, we conducted a series of SEM studies (Figure 3). This indicated that the corneal endothelial monolayer was severely disrupted in the central portion of the cornea beneath the site of the cryoprobe injury. Injured cells tended to become damaged and/or disassociated from each other, whereas noninjured cells adjacent to the area of damage exhibited classic hexagonal endothelial cell morphology. Interestingly, there appeared to be two transition zones between healthy nonfrozen cells and the more centrally damaged ones. The immediate transition at the inner edge of the morphologically normal cells was fairly abrupt; and in some cases, this was on a *μ*m scale (Figures 3(e), 3(f), and 3(g)). More centrally, there was evidence of cellular dissociation (Figure 3(e)) and also total removal of large areas of frozen endothelial cells, exposing a bare Descemet's membrane (Figures 3(d) and 3(h)).

**Figure 4:**
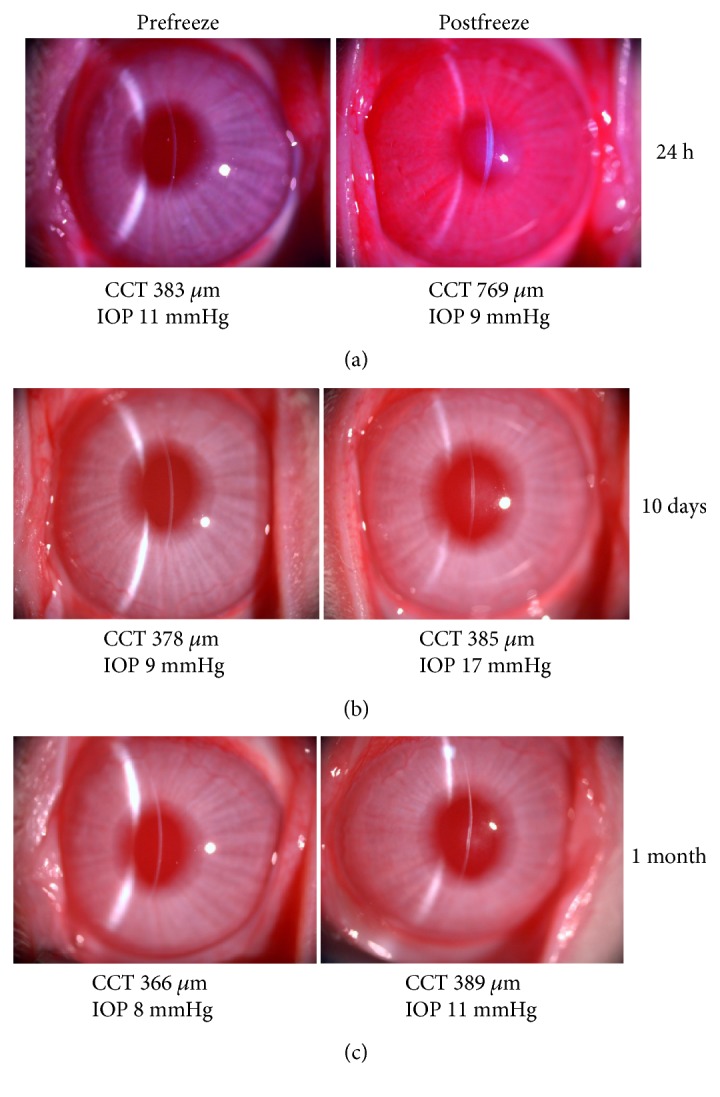
Effects of a 3 sec freeze on the rabbit cornea in vivo using the 2.4 mm diameter/concave profile cryoprobe tip. (a) 24 hrs after freeze, central corneal thickness (CCT) is increased considerably (769 *μ*m) compared to that before freeze (383 *μ*m), and the cornea is hazy, indicative of corneal endothelial damage, as well as epithelial and stromal cell damage. (b) 10 days after a freeze injury (in a different animal), CCT was at normal levels. (c) This was the case also, 1 month after treatment. (b and c) Some corneal haziness at the level of the posterior stroma or Descemet's membrane is evident at 10 days and 1 month.

To investigate endothelial healing after transcorneal freeze, a small number of rabbit corneas were studied. For these investigations, a 2.4 mm diameter/concave profile cryoprobe was used rather than the 3.4 mm concave one owing to the relative thinness (approximately 350–400 *μ*m) of the rabbit cornea compared to that of the pig (approximately 660 *μ*m [29, 30]). This revealed that one day after a 3 sec surface freeze, the rabbit cornea had become significantly edematous, with its thickness approximately twice the normal value (Figure 4(a)). The central corneal thickness returned to normal values by day 7, and this was maintained up to one month postfreeze (Figure 4(c)). Slit-lamp images showed some evidence of corneal haze at the level of the posterior stroma or Descemet's membrane at 10 days and 1 month (Figures 4(b) and 4(c), resp.).

**Figure 5:**
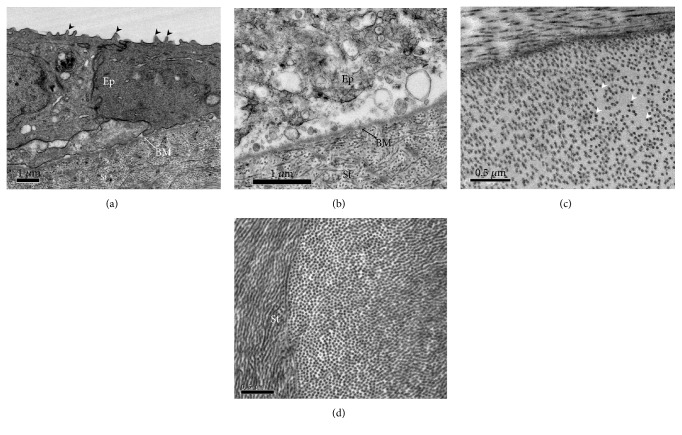
TEM of the corneal epithelium (Ep), epithelial basement membrane (BM), and stroma (St) following a 3 sec transcorneal freeze injury using a 2.4 mm diameter/concave profile cryoprobe on rabbit cornea in vivo. (a) The peripheral epithelium away from the wound zone, 24 hrs after the cryoprobe was applied and appeared morphologically normal. Arrowheads indicate microvilli on apical surface of epithelial cells. (b) Intact basement membrane is observed in the central freeze-injured area 24 hrs postfreeze. (c) After 24 hrs freeze injury, occasional focal regions of stromal matrix disruption were evident in the cornea, manifesting as tissue regions with increased spacing between collagen fibrils. (d) One month after the freezing, throughout the cornea, the spacing between collagen fibril appeared normal. Scale bars, 1 *μ*m (a and b) and 0.5 *μ*m (c and d).

TEM examinations of rabbit corneas 24 hrs after freeze indicated that the corneal epithelium peripheral to the wound area was structurally normal, with typical epithelial stratification, cell-cell contact and surface microvilli (Figure 5(a)). As expected, the corneal epithelium was severely damaged in the central freeze-injured region of the cornea, with considerable cellular vacuolation and membrane destruction (Figure 5(b)). However, it was clear that the epithelial basement membrane remained intact, which presumably is important to aid subsequent epithelial resurfacing of the wound area. In the deep stroma, increased collagen fibril spacing accompanied by disorder in the fibril arrangement was sometimes observed focally 24 hrs postfreeze (Figure 5(c)), but this had been resolved by the 1-month timepoint at which time the stromal architecture appeared normal throughout the cornea (Figure 5(d)). These structural matrix changes likely contribute to the increased corneal thickness and opacity seen at 24 hrs (Figure 4(a)).

**Figure 6:**
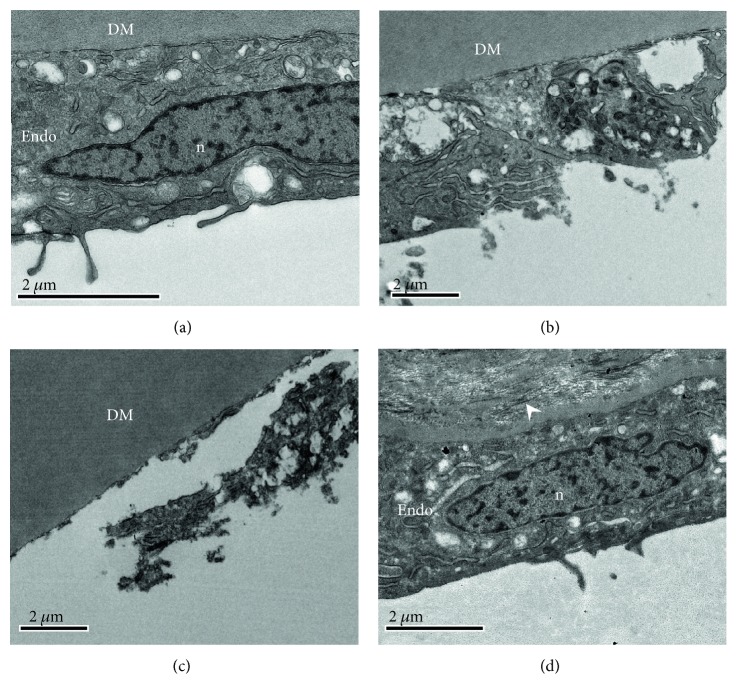
TEM of corneal epithelium following a 3 sec transcorneal freeze injury using a 2.4 mm diameter/concave profile cryoprobe on the rabbit cornea in vivo. (a) An endothelial cell (Endo) at 24 hrs postfreeze in a region peripheral to the freeze-injured area appeared morphologically normal, with normal organelles and nucleus (n). It adhered to Descemet's membrane (DM). (b) Closer to the region below the cryoprobe surface application, there were clear signs of cell damage including the destruction of the cell membrane, while even more centrally, (c) the cell damage was more extreme revealing a bare Descemet's membrane, consistent with the SEM analysis (Figure 3). (d) One month after the transcorneal freezing was performed, the central region of the inner cornea contained fairly normal endothelial cells that were sometimes accompanied by extracellular matrix material (white arrowheads) in the area posterior to Descemet's membrane. Scale bars, 2 *μ*m.

Just as with the corneal epithelium, the corneal endothelium in the periphery of the cornea away from the region of the tissue under the surface wound zone remained unaffected 24 hrs after transcorneal freeze of the central rabbit cornea (Figure 6(a)). The endothelium more centrally, however, began to exhibit clear signs of damage (Figure 6(b)). The central endothelium was often fully debrided with a bare Descemet's membrane that showed no apparent structural changes (Figure 6(c)). One month after freeze, the central corneal endothelium had reattained its normal character, although occasionally fibrous tissue deposition between Descemet's membrane and the recovered endothelium was observed (Figure 6(d)), which perhaps contributes to the deep stromal haze seen at this time (Figure 4(c)).

## 4. Discussion

A number of investigative surgical procedures, which utilize the selective ROCK inhibitor, Y27632, to combat FECD and bullous keratopathy are under investigation, including cell-injection therapy [10–14]. However, one alternative approach for FECD, especially in its early stage, involves freezing the central cornea using a cold probe to damage corneal endothelial cells beneath the surface contact area; this is then followed by the short-term delivery, for one week, of Y27632 in eye drop formulation [10–12]. In these surgeries, freezing was achieved by touching the corneal surface with a stainless steel rod, which had been immersed in liquid nitrogen. An arbitrary freezing time of 15 sec was chosen for these experiments, along with a 2 mm diameter for the steel rod. Encouragingly, the outcomes of these surgeries showed some promise, especially if the extent of the corneal endothelial dysfunction was not widespread, but if the approach is to be adopted more widely by the ophthalmic community, the corneal freeze would probably need to be achieved in a more reliable manner and with more knowledge of the nature and extent of the freeze damage.

Historically, and up to the present day, corneal freezing has been carried out by a variety of methods, most of which use it to induce an experimental injury for research into corneal wound healing [15–17, 28]. Freezing studies tend to employ either a brass rod or dowel, which had been immersed in liquid nitrogen [17–21] or similarly cooled steel ones [22, 23]. Retinal cryoprobes have also been used in investigational studies of transcorneal freezing [24, 33]. Here, we report the design and manufacture of a corneal cryoprobe that uses circulating nitrous oxide as a cryogen and report the type of freeze damage it induces.

Typically, the tips of cryoprobes that use high-pressure gas as a cryogen are made of stainless steel owing to the need to contain high-pressure gas safely. In our design, however, the use of stainless steel would have resulted in a freeze that started in the center of the tip and thereafter spread, albeit quickly, to its outer circumference at a speed which relies on the thermal conductivity of the metal. To achieve a more uniform cooling across the probe tip, we manufactured a cryoprobe tip out of silver, which has a higher thermal conductivity than that of steel. The benefit of this design feature is that the diameter of the freeze in the cornea is decoupled from the depth of the freeze. Cooling in this design is based on the expansion and internal recycling of nitrous oxide inside the cryoprobe tip. At the point of its transition from liquid to gas, nitrous oxide exists at a temperature of −88.5°C, and this transition, which occurs inside our probe tip, of course, rapidly cools it. Owing to thermal conductivity within the whole probe and thermal loss at ambient room temperature, an equilibrium is reached, which in our design means that the temperature at the outer surface of the probe tip reaches −50°C.

The response of a cell to freezing is explained by Fraunfelder in his comprehensive review of corneal cryotherapy, the main mechanisms of cell damage being a piercing of the cell membrane by ice crystals or the creation of a sizeable osmotic imbalance between the inside and outside of the cell because of the removal of liquid water into the ice crystals [34]. Armitage also describes, from the other side of the coin, how careful freezing using time-mediated freeze-thaw protocols accompanied by the use of cryoprotectants can lead to cell survival [35]. The freezing we achieve here can probably be thought of as being fairly conservative in terms of the rate of endothelial cooling, but our current observations clearly indicate that sufficient levels of endothelial damage are achieved after a single freeze treatment. Experiments that applied additional treatments to the same surface location did not enhance the extent of the freeze injury (data not shown). We also found that if the foot switch was depressed to initiate cooling of the probe tip before it was brought into contact with the corneal surface, then no appreciable endothelial freeze damage was seen, even if the cryoprobe was kept in contact with the cornea for periods up to 15 sec. It is not immediately clear why this is the case, but perhaps, frosting of the probe tip when it is cooled in the moist air could contribute to this. This likely lack of frosting also might help facilitate the easy release of the probe tip from the corneal surface after the foot switch is released, which we found to happen within a second or two of the cryogen circulation being suspended.

In the experiments described here, the central corneal epithelium was destroyed by the application of the cryoprobe, as we would expect. But, it is of potential importance that the corneal epithelial basement membrane remains intact as seen by TEM. The lack of epithelial basement membrane damage will presumably aid the epithelial resurfacing of the debrided epithelial area. Fibril arrangement changes are also apparent focally in the stromal matrix after the in vivo rabbit freeze injury, but these are transient, and the increased collagen fibril separation and disorganization which are seen 24 hrs after the treatment subsequently decrease as corneal thickness reduces. The obvious conclusion is that the recovering endothelium is mostly responsible. Of course, the in vivo healing studies reported here do not reflect the situation in the human cornea because of the different behavior of the endothelial cells and their limited replicative ability in humans. TEM also discloses the presence of fibrotic extracellular matrix tissue in the region of Descemet's membrane one month after freeze injury (Figure 6(d)). This might contribute to the deep stromal haze seen at this time, although a potential contribution to light scatter by freeze-damaged keratocytes cannot be discounted.

As mentioned, the use of ROCK inhibitors has aided the recovery of the corneal endothelium in situations where diseased corneal endothelial cells have been scraped away surgically [13] or frozen with a cooled steel rod applied to the corneal surface [10–12]. A report by Balachandran et al. [36] and Shah and associates also suggests that corneal endothelial cells can repopulate in FECD patients after damage alone [37–39]. The data presented here show that endothelial cells can be functionally damaged and/or removed by the application of a cryogenic cold probe and that this can be done in a targeted and reproducible manner with cell damage restricted to the area below the surface contact. Of course, the transcorneal freezing technique is unlikely to induce any significant change to the guttae which exist in FECD and their continued presence will conceivably hinder the reformation of a normal endothelial layer. Nevertheless, cell damage can be achieved through use of a silver 3.4 mm diameter cryoprobe with a concave profile, which was discovered to be the optimal design of the cryoprobes tested in the experiments described here. It thus has the potential to rapidly and reliably induce damage to the human corneal endothelium via transcorneal freezing, leaving the epithelial basement membrane intact. This has the potential to be used prior to the application of ROCK inhibitors to the eye in the form of eye drops [10–12] or as slow-release chemicals from thin films [40] to aid the recovery of corneal endothelial function.

## Figures and Tables

**Table 1:** Data summary of transcorneal freezing for 3 sec on porcine eyes.

Probe tip (mm)/profile	Number of eyes	Number of eyes with successful freeze (%)	Mean/SD damaged area (mm^2^)	Mean diameter (mm)	Mean/SD corneal thickness (nm)
1.8/flat	102	14 (14)	0.79/0.4	1.0	642/64
2.4/flat	108	78 (72)	2.12/1.0	1.6	650/71
2.4/concave	108	76 (70)	2.29/1.0	1.6	645/87
3.4/concave	108	90 (83)	6.91/1.9	2.9	654/59
